# Profiled Wet Spinning of Polyurethane Composites for Soft Dry Electrodes in Transcutaneous Stimulation Applications

**DOI:** 10.3390/ma19030557

**Published:** 2026-01-30

**Authors:** Alexander V. Shokurov, Ee Qing Tee, Abigail Vogel, Gabriel Gmünder, Kai Röllin, Olivier Lambercy, Dane Donegan, Paulius Viskaitis, Carlo Menon

**Affiliations:** 1 Biomedical and Mobile Health Technology Laboratory, Department of Health Sciences and Technology, ETH Zurich, Lengghalde 5, 8008 Zurich, Switzerland; 2 Rehabilitation Engineering Laboratory, Department of Health Sciences and Technology, ETH Zurich, Gloriastrasse 37/39, 8092 Zurich, Switzerland; eteeee@student.ethz.ch (E.Q.T.); lgmuender@student.ethz.ch (G.G.); kroellin@student.ethz.ch (K.R.); olivier.lambercy@hest.ethz.ch (O.L.); dane.donegan@hest.ethz.ch (D.D.)

**Keywords:** wet spinning, polyurethane, composite, piezoresistive, conductive composite, transcutaneous stimulation, dry electrodes

## Abstract

Transcutaneous electrical nerve stimulation techniques (TENS) are rapidly gaining attention for their potential in various clinical applications. One such technique is transcutaneous auricular vagus nerve stimulation (taVNS), and it involves delivering nerve stimulation through the skin of the external ear. However, taVNS relies on electrodes that must conform to the complex anatomy of the ear while maintaining stable electrical performance. Conventional taVNS electrodes, typically rigid metal or adhesive pads, are uncomfortable, difficult to position, prone to drying, and costly to produce. Here, we present and evaluate two complementary fabrication approaches for soft dry electrodes suitable for taVNS, which are compliant with curved anatomical features and can be operated without gel. The first employs wet spinning of a conductive elastomer into fibers, while the second extends this method to create hollow cylindrical geometries. The resulting spongy polymer composite electrodes exhibit tunable geometry, high conductivity, mechanical resilience under strain and compression, and low material impedance confirmed through bench and human testing, even under dry conditions. These properties are critical for in-ear and broader transcutaneous stimulation applications, highlighting the potential of these fabrication methods for next-generation soft bioelectronic interfaces.

## 1. Introduction

Transcutaneous electrical nerve stimulation (TENS) is increasingly recognized as a powerful, non-invasive means to modulate neural activity for therapeutic applications [1]. One such application is transcutaneous auricular vagus nerve stimulation (taVNS), which excites the vagus nerve by delivering electric pulse through the skin of the ear, particularly the cymba and cavum conchae [2]. taVNS is an active area of research for its possible application in neurorehabilitation [3,4,5].

The main hurdles in the operation of existing taVNS devices are as follows: inconsistent skin contact between the electrode and the ear, lack of adaptation to anatomical variability in auricular anatomy, and lack of standardized, user-friendly wearable electrodes [6,7]. In simpler terms, the electrode of the device needs to be comfortable to wear in-ear, while also forming good electrical contact through the skin interface. But human ears have very complex anatomy; no two ears are the same. Utilization of very compliant and soft electrodes can ensure good conformity to various ear anatomies while maintaining user comfort.

Many commercial VNS devices utilize clip-style electrodes, which are not very comfortable for users [7]. Novel, non-metallic electrodes (e.g., clips) include conductive hydrogel-based electrodes that mimic the performance of traditional wet electrodes but are able to hold a shape complementary to the ear anatomy; these hydrogels, however, are prone to drying and need frequent replacement [8,9,10]. Another form of electrode, that is considered non- or minimally invasive, is the microneedle array, which is designed to gently penetrate the stratum corneum to reach the epidermis, thus lowering through-skin impedance [11]. State-of-the-art in non-invasive electrodes is represented by textile conductors [12], ‘tattoo’ patches [13], and ion-conducting polymers [14]. While these show excellent properties and performance, they remain rarely used in taVNS applications mainly due to the complexity of compliance with the non-planar ear anatomy and lack of a user-friendly application (or solution).

An ideal ergonomic ear electrode should fit snugly within the cavities of the external ear, which corresponds to cm scale lengths and mm scale curvatures, but more importantly, they need to adapt and comply with a wide variety of anthropometric variations of the ear [15,16]. At the same time, these soft and compliant electrodes should exhibit minimal skin–electrode contact point impedance to ensure the best stimulation performance [17].

Most intensive research in the context of innovative adaptable conductive electrode materials is carried out in the domain of conductive elastomer composites [18].

Conductive elastomer composites are elastic, stretchable, and flexible materials made of an elastomer matrix. This elastomer matrix provides the mechanical properties (e.g., shape-forming and elasticity), and the conductive filler provides the electrical (or ionic) conductivity, potentially together with plasticizers, inert fillers, functional cross-linkers, etc. Several stretchable conductive elastomer materials have been developed specifically for conformal transcutaneous stimulation based on silver nanowire–silicone materials, showing good strain–stress properties [18], carbon nanotube–silicone composites with excellent conductivity [19], sustainable polyurethane and silver flake composites [20], to name a few.

Polyurethane (PU) composites are considered one of the most promising materials from the standpoint of both the operational qualities needed for taVNS (adaptability and softness at application point, electrical conductivity) and aspects such as sustainability, biocompatibility, and cost [19,21]. PUs are versatile polymers formed from isocyanate-based hard segments and polyol-derived soft segments. By adjusting the polymer structures, the PU structure and performance can be finely tuned for a wide range of applications. They are known for their remarkable mechanical and surface qualities, such as flexibility, high stretchability, strong impact and tensile performance, resistance to wear, durability in outdoor conditions, and stable appearance with long-lasting gloss and color [21]. There are many methods to produce PU composites and to shape them into VNS-capable electrodes. In the case of thermoplastic polyurethanes, melt compounding and extrusion remain the most popular techniques. In both methods, PU pellets and conductive filler are mixed together at elevated temperatures and then pressed out through an aperture to form a sheet, filament, or granules [22,23]. This powerful, scalable method is characterized by some notable challenges related to high-temperature degradation of the polymer, even distribution of conductive fillers, and, most importantly, geometry. Melt-extruded parts are generally limited to simple cross-sectional profiles (films, tubes, fibers). Producing complex 3D shapes or micro-features requires additional downstream processing (e.g., overmolding, machining), reducing design flexibility.

Another method is solution casting. In this method, conductive fillers are dispersed in a PU–solvent mixture, cast into films, before the solvent is evaporated and the PU is fully cured. Making composites this way is simple in processing, allows for easy introduction of additional components (such as functional crosslinkers), and allows for precise thickness and geometry control [19,24]. Although it is a ‘wet’ method, it also involves production challenges such as residual solvent, long processing times, and difficulty in creating 3D geometries. Several groups have also exploited compression and injection molding to produce PU-based conductive pieces with device-ready form factors, which can potentially operate as electrodes [20,25,26].

The methods described above tend to produce bulk PU composites, meaning that the filler and matrix are homogeneously mixed and formed into electrodes. In this configuration, the elasticity and hardness of the composite polymer are the only factors that control how compliant it can be. Notably, the addition of conductive filler to achieve electrical conductivity almost always negatively affects the mechanical properties of the composite, making it stiffer, more brittle, lower in tensile strength, and with lower elongation-to-break resistance [26,27]. This is the main motivation in research involving bleeding-edge fillers like silver nanowires and carbon nanotubes, the weight loading of which needs to be much lower compared to traditional fillers (graphite, metal powders) to achieve the same conductivity, without as much loss of mechanical properties. Meanwhile, carbon black remains cheaper than the nanotubes/nanoparticles in terms of achieving a similar electrical performance of composites and devices [28,29] and is known to be safe to use with human skin [30], which is relevant for TENS applications. Compared to nanotubes and nanoparticles, a higher concentration of carbon black is needed to achieve similar electrical performance, which also leads to much stiffer composite materials.

However, additional flexibility can also be achieved by foaming the composite, creating a large number of pores within the structure. This method can make a material, which is relatively stiff in bulk, much more compliant owing to the foam/sponge geometry. Spongy PU composites are usually produced by the injection of gas into the composite at the stage of formation [31] or by post-treatment of pre-foamed PU, such as impregnation by conductive material [32,33]. Apart from PU structures, porous materials are studied for applications in ultrasoft and conformable electrodes [34,35].

Flexibility can also be achieved by forming lower-dimensional objects, such as thin films and fibers. One of the techniques to form composite elastomeric fibers is wet spinning. In this process, the composite material precursor solution, which is called a spinning dope, is run through a bath that coagulates/precipitates the solution into a solid fiber that is then spooled and drawn. Intriguingly, wet-spinning, a process traditionally used to form 1D PU materials, i.e., long fibers for applications in (smart) fabrics and textile sensors, also leads to a somewhat spongy/foamy internal structure on the micro-level, producing lightweight, soft, and compliant fibers [36,37].

In this study, inspired by the PU wet spinning process, the aim is to use the same chemistry and procedures as employed in fiber production, but to produce 3D geometries with more complex cross-sections that could be ideally suited for the development of compliant electrodes, as required for taVNS applications. For this, a conductive composite based on carbon black and polyurethane was produced and then shaped into a soft cylindrical hollow electrode using the ‘profiled wet spinning’ technique described here. These spongy conductors are then tested for mechanical and electrical properties in the context of transcutaneous stimulation applications, to demonstrate their potential for use as dry soft electrodes in the external ear.

The main aim of this profiled wet spinning technology is to provide a means for producing versatile and adaptable electrodes based on conductive elastomeric composites. Ideally, the produced electrodes should have custom shapes and enough mechanical flexibility and elasticity to conform to the complex ear anatomy, while also providing minimal skin contact impedance for stimulation (ideally in the kΩ range [38]).

## 2. Materials and Methods

### 2.1. Materials

Polyurethane (PU) of the Elastollan EG1170A brand was acquired from BASF (Basel, Switzerland). Before dissolution and further processing, the PU pellets were oven-dried in an open container at 80 °C overnight.

DMF (anhydrous, 99.8%) was acquired from Sigma-Aldrich (Buchs, Switzerland) and was dried using 4 Å molecular sieves also procured from Sigma-Aldrich.

Carbon black (CB)—Vulcan^®^ XC72, powder with ~250 m^2^/g porosity and bulk density of ~0.265 g/cm^3^ was acquired from Sigma-Aldrich (Buchs, Switzerland) and used as received.

### 2.2. Preparation of Conductive PU Composite

The conductive polymer composite used in this study consists of PU and CB. As a precursor of the (profiled) wet-spinning process, these two components were dissolved in dry dimethylformamide (DMF). Briefly, the spinning dope solution was formed by dissolving PU pellets in anhydrous DMF (15% *w*/*v*) under vigorous stirring overnight with moderate heating (50 °C). Note: PU pellets should be added in portions to prevent clumping.

To impart electrical conductivity to the composite, the PU solution was mixed with CB using a Thinky ARV-310P (Tokyo, Japan) planetary mixer at 2000 rpm. A range of filler ratios from 4 to 10% *w*/*w* filler to polymer solution was investigated. After mixing, the spinning composite solution was either used for spinning immediately or re-mixed using the same procedure just before spinning. The final carbon black–polyurethane composite (CBPUC) was then obtained by (profiled) wet spinning into a coagulation bath containing distilled water, as described below.

### 2.3. Wet-Spinning and Profiled Wet-Spinning Extrusion Technique

Conductive CBPUC fibers were formed by wet spinning into a distilled water coagulation bath. To do this, the CBPUC spinning dope was loaded into a syringe equipped either with a Luer-lock and/or 14-gauge needle. Loaded syringes were then either centrifuged or degassed under low vacuum (0.2 kPa) before extrusion. This step prevents bubbles in the extrusion line, which would negatively affect the sample quality. For ‘profiled wet spinning extrusion’ of complex shapes, as required for the target application in taVNS electrodes, custom nozzles were produced using Original Prusa SL1S Speed Printer (Prusa Research a.s., Prague, Czech Republic) with a tough resin (Prusament Resin Tough, also manufactured by Prusa Research a.s.). Smaller nozzles can be designed to fit the Luer-lock connection of the syringe, while larger nozzles need to be fitted directly over the syringe barrel to allow for larger flow.

**Figure 1:**
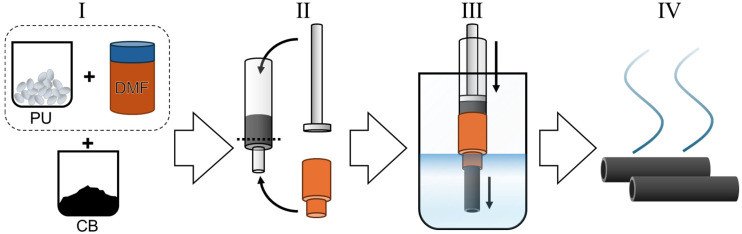
Schematic of the profiled wet spinning process to form electrically conductive CBPUC fibers/hollow cylindrical electrodes. In stage I, the spinning dope is formed: polyurethane pellets are dissolved in DMF overnight; the resulting solution is then loaded with conductive CB filler using planetary mixing. In stage II, a syringe is loaded with the spinning dope, centrifuged to remove air bubbles, and equipped with a plunger and a nozzle of the desired shape. The dashed line symbolizes cutting the front of the syringe off in case of larger nozzles that fit directly over the syringe barrel. In stage III, the spinning dope is slowly pushed through the nozzle into a distilled water coagulation bath, where the polymer with filler precipitates, forming an extruded shape, the cross-section of which depends on the nozzle shape. In stage IV, the produced shapes are left to solidify in the coagulation bath for a prolonged time (1–3 h) and subsequently taken out to dry in air at room temperature overnight. After drying, the soft electrodes are ready to use.

The syringe equipped with either a needle or nozzle was then loaded into a syringe pump, and the needle/nozzle was lowered into the volume of distilled water (much larger than the volume used in extrusion, typically >1 L). It is important not to disturb the water during the ongoing extrusion, as any movement of the bath would affect the shape at the extrusion point. Flow rate varied depending on the needle/nozzle used, e.g., 10 mL/h for the wet spinning using gauge 14 needle and 30 mL/h for the larger profile nozzles. The spinning dope was extruded into the water, where it solidified to form a mass with the desired shape—either a fiber or a shape with more complex cross-section geometry. If removed from the bath immediately, the shape and structure of the fiber/shape are not preserved, as the DMF/water exchange is not that fast. To ensure the absence of residual solvent, the produced fiber/hollow cylindrical electrode is left in the coagulation bath for 1 h for fiber-form samples and 3 h for the thicker electrodes. The samples produced were then dried in air at room temperature overnight (at least 8 h for the thicker samples). The entire process is illustrated in Figure 1.

### 2.4. Electromechanical Testing

Electromechanical studies involving tensile and compression tests were carried out using a Zwick/Roell electromechanical tester machine (Zwick Roell, Ulm, Germany), while the electrical properties of the sample were measured via Hioki IM 3636 LCR meter (Hioki E.E. Company, Nagano, Japan). Studied samples were fixed in the machine using custom-built 3D printed fixtures (produced in the lab).

In tensile testing, strain was applied to the sample in a cyclical fashion, repeating the application of the desired strain and moving back to 0% strain every cycle, usually at frequencies of 1–2 Hz. Electrical connections with the measurement setup were made at the ends of the fiber being tested using alligator clips.

In compression tests, the sample was placed on a conductive bottom plate made of aluminum. The actuated part of the compression test machine was instrumented with a pusher covered by conductive copper tape. The pusher and the bottom plate thus formed a pair of electrical contacts used to measure the resistance of the sample during compressive tests. Compression was performed either in a stepwise manner, controlled by position, i.e., advancing 0.1 mm at a time, or in cyclical tests controlled by force. i.e., compressing the sample until the desired force is reached. 

### 2.5. DMF Retention Tests

A Bruker Alpha II Fourier Transform Infrared (FTIR) spectroscopy setup (Bruker Corporation, Billerica, MA, USA) was used to establish the retention of the initial solvent DMF after the extrusion of the samples. ~1 cm length of samples taken from the coagulation bath at different times and dried overnight, as per the usual procedure described above. The samples were then shredded into smaller pieces and extracted with acetone (~2 mL) by mechanical agitation for 10 min. A droplet of such acetone extract was placed on the FTIR spectrometer sensor and left to evaporate, after which the spectrum was recorded. Acetone containing 10% DMF was used as positive control, and pure acetone as negative control.

### 2.6. Evaluation of Electrode Impedance Characteristics

**Figure 2:**
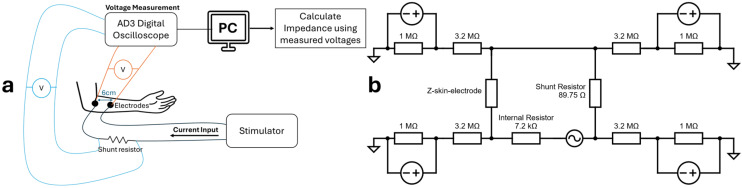
(**a**) Schematic illustration of the experimental electrical setup and its placement on the participant’s arm for impedance measurements, and (**b**) the electrical circuit diagram representing the setup.

To characterize the electrical properties of the custom electrode material intended for the taVNS device, two complementary approaches were used: frequency–domain impedance spectroscopy and time–domain impedance estimation under clinically relevant stimulation. Both dry and wet (Parker Signaspray, produced by Parker Laboratories, Inc., Fairfield, NJ, USA) electrode conditions were evaluated in both approaches. Custom-made electrodes were compared against flat silver metal sheets, representing more conventional transcutaneous stimulation electrodes. Impedance spectroscopy was performed using the Analog Discovery 3 (AD3, Digilent Inc., Taiwan, China) in impedance analyzer mode, which applied sinusoidal signals (100 mV for skin–electrode measurements) across a sweep from 1 Hz to 1 MHz. Electrodes were mounted with a constant spacing of 6 cm and pressed against the skin using a weighted fixture (380 g per electrode) to replicate tight-fitting electrode-ear conditions. The arm was used in our preliminary stimulation studies as a flatter anatomical site, allowing consistent electrode spacing and inter-participant variability. While not representing the ear geometry, such an experimental setup allowed us to initially disregard anatomical complexity during early development, improving test reproducibility. This protocol allowed assessment of the frequency-dependent behavior of the polymers and the skin–electrode interface, with particular emphasis on 25 Hz, corresponding to the stimulation frequency typically used in taVNS [7,39]. The series resistor value was adjusted based on the skin’s condition: a 100 kΩ resistor was used for dry skin, and a 10 kΩ resistor for wet skin. The overall setup in terms is illustrated in Figure 2.

In parallel, the AD3 was used in oscilloscope mode to evaluate impedance during biphasic current-controlled pulses delivered by an in-house developed, current-controlled taVNS stimulator at standard parameters (charge-balanced, rectangular biphasic pulses, 250 µs per phase, delivered at 25 Hz). The stimulator provided currents in 100 µA increments, starting at 100 µA and increasing until the participant’s tolerance limit was reached, with a compliance voltage of 80 V and a current limit of 5 mA. A shunt resistor (98.75 Ω) was inserted in series to measure actual current flow, while voltages across both the shunt and the electrode interface were simultaneously recorded. Impedance was derived pulse-by-pulse from Ohm’s law, using the average current during the plateau of the first phase. For each stimulation intensity, ten pulses were acquired, with improperly captured signals discarded. Testing was approved by ETH Zurich Ethics Commission (24 ETHICS-027, informed consent was obtained from all subjects involved in the study), and after providing informed consent, data were collected from 8 participants. For impedance analysis, a maximum stimulation current of 3 mA [40] was selected as a clinically relevant limit for taVNS. Given the stimulator’s maximum output of 80 V, this corresponds to a theoretical upper impedance limit of approximately 26.7 kΩ, which was used to define the operational range for evaluating electrode performance.

## 3. Results and Discussion

### 3.1. Preparation of CBPUC and Fiber Wet-Spinning

In traditional wet spinning, the spinning dope (spinning solution) is passed through a needle or a spinneret into the coagulation bath, where it solidifies and forms a fiber. If PU dissolved in DMF, pure water can be used as a coagulation agent, as it will draw the solvent from the dope and precipitate the polymer. To test if this process can be used to produce a thicker conductive elastomer fiber, a spinning dope consisting of PU solution in DMF with varying weight percentages of CB was produced. This dope was then loaded into a syringe equipped with a gauge 14 blunt needle. By submerging the needle in water and pushing the spinning dope through at a constant rate of 10 mL/h with a syringe pump, a continuous elastomeric conducting fiber with a diameter of ~1 mm was produced. The produced fibers were then left for 1 h in the water to complete the solvent exchange, i.e., diffusion of DMF from the fiber into the water. After that, the fibers were left to air-dry. At this point, the fibers were considered ready to use. Conductive and elastic CBPUC fibers were produced this way from spinning dope containing 2, 4, 6, 8, and 10% *w*/*w* of CB. The CB filler loads here are reported as the weight of CB over the PU/DMF solution weight. After DMF removal through the spinning process, the CB-to-PU weight ratios translate to 13.3, 26.6, 40, 53.3, and 66.6% *w*/*w*, respectively. Notably, these are relatively high loadings, compared with the state-of-the-art composites that use nano-carbons and other advanced materials, where the loading of conductive filler per polymer weight is usually <10% [41]. However, CB is a more readily available material compared to nano-carbons and other conductive nanomaterials.

In order to establish if the produced material can be used for electrical applications, including TENS, we assessed its electrical conductivity. In a simple test, the resistance of the fibers was measured lengthwise multiple times within several samples. The average data for fibers produced with various filler contents are reported in Table 1. The table also presents the results of mechanical testing of the samples upon strain.

The samples produced with 2% CB spinning dope, while conductive, did not exhibit high electrical conductivity, most likely due to too low a concentration of CB in the material, precluding it from the formation of particle percolation networks required to conduct electricity. This phenomenon is well-known in composites with conductive fillers: the concentration of the conductive filler particles needs to be such that the filler particles can meaningfully interact with each other within the composite to provide a path for the electricity to follow. Usually, additional conductive filler at lower concentrations does not lead to significant improvements of the overall composite conductivity until this concentration, called the percolation threshold, is reached; after which the conductivity increases by several orders of magnitude due to the establishment of the conductive percolation networks within the composite [18,42,43].

Indeed, spinning with 4% CB content led to a composite fiber exhibiting almost fivefold improved conductive properties. Unfortunately, these low CB content samples, while very stretchable, proved to be highly resistive with an average resistance over 100 kΩ/cm along the length of the fiber. On the other hand, samples with relatively high loading (>6%) of the filler were excellent conductors with resistances below 1.5 kΩ/cm. While the 8% sample was stiffer and tended to show some plastic deformation, the 10% sample was found to be brittle, showing significant fragmentation when handling and exhibiting almost no elastic behavior. Such mechanical properties would make the composite unusable as an electrode for transcutaneous stimulation, as they need to be elastic and durable.

On the other hand, wet spinning of the 6% CB dope produced a well-conducting (at 1.5 ± 0.17 kΩ/cm), soft, and mechanically robust fiber, capable of elongation up to 55% before breaking. In terms of conductivity, the best composite should ideally be as conductive as possible. While in TENS applications, loss of current is inevitable, it is expected to occur on the skin interface and not the composite. This means that as long as the electrode has lower impedance than skin, it has potential use in TENS; this is usually assumed to be around 20 kΩ [44]. While the resistance can be lowered at higher CB content, improvement of conductivity with filler loads higher than 6% CB are not as significant, while mechanical properties decrease. By simply assessing the ratio of electrical conductivity to maximum elongation, we have chosen the 6% CB composite to be the most promising for further application in spinning. Thus, 6% CB spinning dope was used in all further tests, unless noted otherwise.

To further assess the electromechanical properties of this wet-spun composite, the electrical resistance at mechanical deformation was measured. Electromechanical properties of the composite itself can also be assessed through tensile tests. Therefore, the performance of the composite was tested by evaluating the stability of its electrical resistance during mechanical elongation.

**Figure 3:**
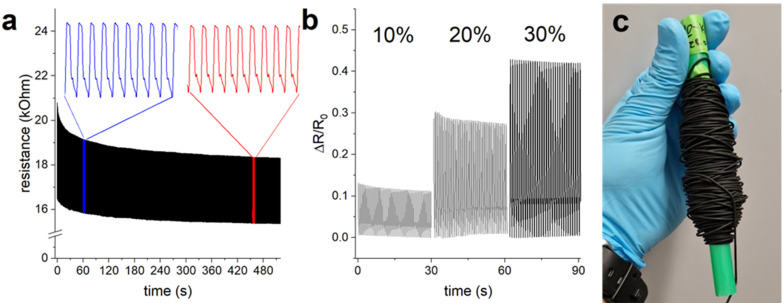
Measurement of resistance of the 6%CB CBPUC fiber produced with a 14-gauge needle tip (**a**) upon cyclic application of 15% strain at 2 Hz for 1000 cycles. The inserts show the close-up of the signal for 5 s in the beginning (blue) and later portions (red) of the test. (**b**) Sensitivity, i.e., change of resistance over initial resistance of a CBPUC fiber upon cyclical application of 10, 20, and 30% strain. (**c**) A photograph of a spool of the tested CBPUC fiber.

In this test, the CBPUC fiber was subjected to cyclic strain testing, where 10% strain was applied to it with a frequency of 2 Hz. Figure 3 shows that the resistance of the fiber changes when stretched; this is a characteristic behavior of piezoresistive materials, in which the conductive percolation network is being thinned out at elongation, thus increasing the resistance [45]. Another noticeable trend is that the overall resistance of the sample drops in the first hundred strain cycles and then stabilizes, with the overall change becoming insignificant (<5%) after 500 cycles. This effect is brought on by the restructuring of the CB percolation network within the composite. This is a well-known pre-conditioning effect in piezoresistive materials, which occurs due to the conductive network rearrangement, and is especially noticeable for composites that have conductive filler concentration close to the percolation threshold [45]. This effect is not always positive, i.e., leading to decreases in resistance, but in our case, pre-strain does result in better conducting material. The resistance of the CBPUC fiber sample improved slightly over the duration of the test of 1000 cycles, with its resistance at rest lowering from an initial 16.5 kΩ to 15.3 kΩ at the end of the test. Moreover, cyclical application of strain up to 30% did not destroy or negatively affect the sample (Figure 3b). The change in the fiber’s resistance during elongation shows its potential to be used as a strain sensor, but that is outside the scope of the present study.

It is known that PU can degrade over time, especially under UV and humidity exposure [46]. In the perspective use case, the taVNS electrodes would not be exposed to any of these factors for a prolonged amount of time, as the envisioned electrodes are meant to be disposable. Degradation of PU in storage, however, is still important. Electromechanical tests and resistance measurements after storage of a fiber in a polyethylene bag and in darkness for 30 days show that the operational parameters (elasticity and electrical resistance) of the fiber do not change significantly.

The developed wet-spun PU fiber exhibits a resistance in the kΩ range, comparable to PU/CB composites, achieving 1–100 kΩ/cm resistivity near percolation [42], and PU/CNT fibers reaching lower values of ~10–1000 Ω/cm at high loadings [47]. Notably, comparison of resistances, especially in fiber form, is tricky, as several methods of reporting it can be used. Also, mechanical properties should be considered alongside the electrical ones when considering TENS applications. All in all, optimization of electrical resistance was not the primary goal of the present study, which focused instead on mechanical and structural properties, as well as the possibility to perform profiled spinning for taVNS electrode applications

### 3.2. Profiled Wet Spinning Extrusion of CBPUC for Electrode Formation

**Figure 4:**
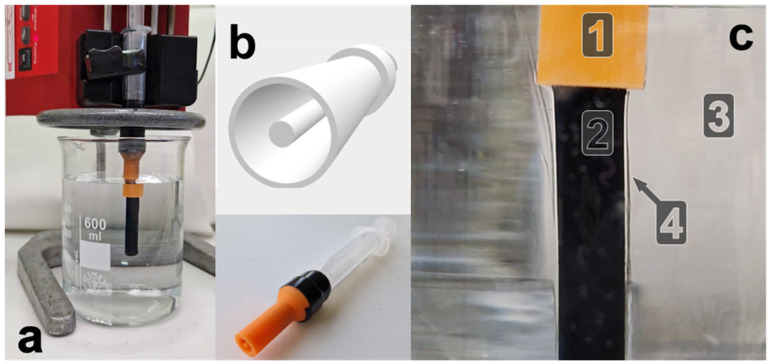
(**a**) Photo of the experimental profiled wet spinning extrusion setup, showing the water coagulation bath, syringe pump loaded with CBPUC spinning dope syringe, equipped with the extrusion nozzle. (**b**) Custom nozzle design and a photo of the nozzle fixed on a syringe. (**c**) Close-up photo of the process of profiled wet spinning extrusion: (1) extrusion nozzle, (2) hollow cylindrical CBPUC electrode being extruded into (3) water coagulation bath, with a visible (4) diffusion front of DMF surrounding the extruded sample and mixing with water.

Although the aforementioned CBPUC fibers were electrically conductive, the resultant small-diameter structure is not directly optimized for the ear anatomy and would require further processing to integrate with the other components of the stimulation device. While it can be wrapped around a support to act as an in-ear electrode, it has no means of incorporating internal cavities for wiring or robust mechanical attachment. To address these limitations, the fabrication process was refined to produce larger, hollow cylindrical electrodes better suited to the anatomical constraints of the ear and more readily integrable into complete stimulation assemblies. The aim was to evaluate whether the wet-spinning method could be adapted to generate such hollow geometries while maintaining the electrical and mechanical performance required for taVNS applications. To do so, the same wet spinning setup was used, but the needle was replaced with a custom nozzle (Figure 4a) that extrudes the CBPUC spinning dope into a desired shape. The nozzle with a large cylindrical cavity (radius of 8 mm) and a coaxial 3 mm pin in the middle was designed (Figure 4b).

By pushing the same spinning dope through the nozzle, the aim is to achieve a thicker product with a hole in the center. This design aims to reduce the amount of polymer consumed (as compared to a non-hollow cylinder), produce a lighter final product, and allow for easier integration with electronics, i.e., the hole in the middle can be used to add wiring or to pull the soft electrode over a rigid part of the device, such as an underlying connector or wiring. This process can now be called a profiled wet spinning extrusion, since the process uses the same chemistry as wet spinning, but produces a sample with a more complex cross-section profile, similar to what is achievable by extrusion.

In the spinning process, the spinning dope is pressed through the nozzle (1) into the distilled water coagulation bath (3) (Figure 4c). This leads to precipitation of the PU and CB from DMF to form a long, solid elastomer mass (2) with a cross-section defined by the nozzle profile. The process of solvent exchange, namely diffusion of DMF into the water, is quite visible, as the diffusion fronts form around the produced sample and propagate as shimmer in the volume of the coagulation water. We have found that at least 3 h in the coagulation bath is required to reliably produce fully solidified electrodes due to the larger size of the shape produced. If the samples are removed from the bath any earlier, the cores remain soft or “mushy”, which is likely caused by retention of DMF.

Analogously, the rate of extrusion plays an important role, with too fast a rate leading to loss of the cross-section shape, e.g., collapse of the hole in the middle or deviation from the intended form. Drying after the profiled wet spinning extrusion is also important. Drying in the oven at elevated temperatures can lead to the expansion of water inside the samples, leading to the collapse of the sponge-like structures. Employment of volatile solvents, such as ethanol or acetone, to replace water and dry more quickly also leads to the loss of form. Hence, we continued with simple air drying at room temperature.

Another important aspect is the shrinkage of the material. While electrodes are being spun/extruded, the overall volume corresponds extremely well to the cross-section. However, during the solvent exchange process, the polymer de-swells by losing DMF. Even more shrinkage occurs upon drying. While the outer diameter of the nozzle cavity was 8 mm, the samples produced through it were around 6.5–7 mm in diameter, while the inner “hole” increased to 2 mm from the intended 1 mm. This represents a reduction of the cross-section of the final product versus the nozzle design of around 30%, which needs to be considered in the electrode design. For example, knowing that the composite shrinks by 30%, the nozzle diameter or size of other features can be increased appropriately to compensate. Other parameters, such as coagulation bath and spinning dope compositions, stirring, and bath exposure time, can drastically affect the shrinkage of the produced shape as well.

### 3.3. CBPUC Electrode Properties

After drying overnight at room temperature, the electrode became completely dry to the touch and passed the tape test, in which adhesive office tape adhered to the sample can be lifted off with no residue. The produced CBPUC tubes are flexible, stretchable, and elastic. This is mostly due to the inherent elasticity of the PU used in this work, but also due to the microporosity of the material. Such a microporous structure forms due to the solvent–water exchange during the wet spinning [36,37].

**Figure 5:**
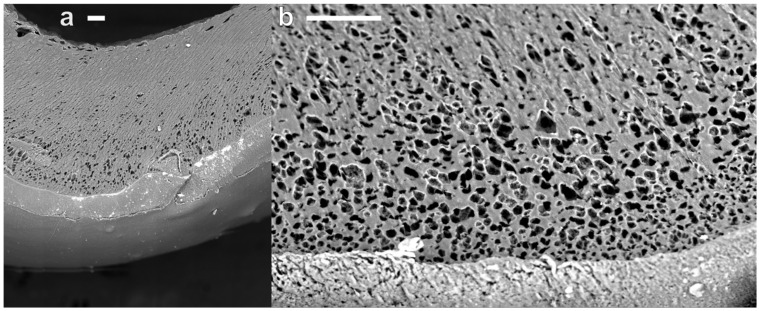
Scanning electron microscopy images of the CBPUC electrode sample. (**a**) Compound image of a part of the cross-section of the electrode, showing outer ‘skin’ part, porous middle part, and large inside cavity formed through profiled wet spinning. (**b**) Close-up image of the porous middle part of the sample, outer ‘skin’ part is visible at the bottom of the image. The scale bar on both images is 100 µm.

In the present case, the extruded cross-section was large compared to the usually <1 mm thick spun fibers, leading to a relatively more complex material structure. Figure 5a shows a scanning electron microscopy compound image of a partial cross-section of a hollow cylindrical CBPUC electrode. The outside part of the profiled wet spun sample exhibits approximately 100 µm thick ‘skin’ comprised of compact material that lacks any large pores. It most likely forms due to a quicker solvent exchange occurring at the spinning dope/water interface. Whereas deeper into the sample, this process is slowed down, and the polymer precipitates, trapping more water in it. When the water is evaporated during drying, the remaining polymer takes on a spongy structure, as can be seen from a close-up image in Figure 5b. The pores inside the material are roughly circular with diameters around 11.38 ± 3.79 µm. These micro-pores provide additional compressibility to the spongy CBPUC electrodes. Most likely, it is indeed the porosity of the wet-spun PU that provides elastic moduli (<1 MPa) much lower than the base bulk PU (~20 MPa).

**Figure 6:**
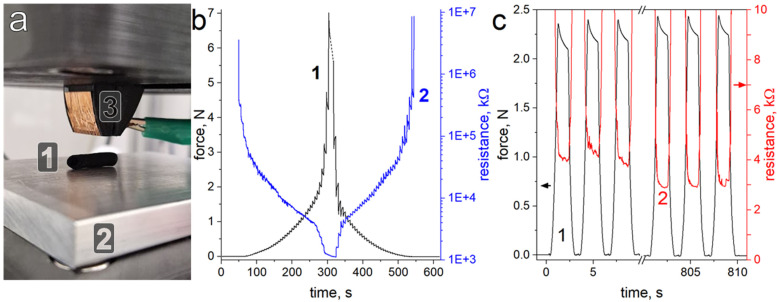
(**a**) Digital image of the setup used for electromechanical compression tests with (1) CBPUC electrode sample, (2) aluminum base plate, and (3) pusher with a conductive surface. Aluminum plate and pusher are connected to the LCR device for resistance measurements. (**b**) The results of the electromechanical compressive measurements performed with the tooling (pusher) approaching and then moving away from the base plate in 0.1 mm steps, showing (1) force exerted by the sample being compressed and (2) its electrical resistance under compression (log scale). (**c**) Cyclical compression tests of the studied electrode, showing (1) force exerted by the sample being compressed to the same degree in every cycle, and (2) its electrical resistance under compression.

Electromechanical compression tests were carried out to confirm that the structure of the material retains good compressibility and conductivity to be used as an electrode in taVNS stimulation. Figure 6a shows a digital image of the compression testing setup, where the CBPUC sample (1) is placed between the aluminum baseplate (2) and the specially designed pusher (3) mounted to the actuated part of the compression tester machine. Both the baseplate and the pusher are connected to the resistance measurement device. When the pusher is disengaged (as depicted in the figure), no electrical contact is formed, and the reading of the measurement setup shows >10 MΩ of resistance.

The compression tester machine is then programmed to slowly close the gap in steps of 0.1 mm, which are held for several seconds, while both the force exerted on the pusher and the electrical properties are recorded simultaneously (curves 1 and 2, respectively, presented in Figure 6b). The data show that the gradual decrease of distance at first does not produce any measurable force (beginning of curve 1) while the tooling does not touch the sample. When the pusher advances enough to form the first electrical contact with the sample (around 80 s, Figure 6b), a small non-zero force is observed, signifying physical contact between the pusher and the CBPUC sample. At the same time, high (in the order of 3 MΩ) but discernible resistance is read by the measurement device, meaning that such a light touch already closes the electrical circuit between the pusher through the composite sample to the aluminum base plate.

Further stepwise closing of the pusher starts compressing the CBPUC electrode, with the force slowly increasing. The electrical resistance of the sample, however, plummets several orders of magnitude when appreciable contact is made (Figure 6b, curve 2). At compression corresponding to around 2 N, the sample resistance drops to around 3–4 kΩ. Further compression (at around 300 s, Figure 6b) leads to a rapid increase of the observed force, coinciding with the folding of the inner tube of the sample and severe compression of outer walls, and single kΩ resistances are observed. However, such a degree of deformation is probably not going to be experienced by the electrode in a taVNS system, as forces above 5 N are considered uncomfortable for dry electrodes and earplugs [48,49].

To see if the produced soft dry electrode can be reused many times, a cyclical compression test was performed. The sample was repeatedly compressed to around 2.5 N, corresponding to a force that is still considered comfortable for in-ear applications [48,49]. Figure 6c shows the results of such testing, with curve 1 showing the force generated at each compression and curve 2 showing the resistance of the sample. The electromechanical properties of the electrode did not change significantly over 300 cycles of cyclical compression. Visual inspection of the sample after the cyclical test also showed no signs of plastic deformation or damage.

Electromechanical investigations show that the shapes produced by continuous CBPUC profiled wet spinning are promising for application as dry soft electrodes in taVNS applications.

### 3.4. DMF Retention by the Produced Material

The materials used in the production of the electrodes are generally considered safe for the skin [30,50], except for the solvent DMF, which is considered a toxic compound even for skin exposure. While it is evident that a prolonged time of 3 h is required for the CBPUC profiled electrodes to solidify, indicating the requirement of DMF leaving the material in solvent exchange, it is crucial to understand the extent this solvent is still present in the final product before application to skin.

To evaluate this, we have employed FTIR spectroscopy, in which characteristic bands of DMF should be easily distinguishable even in very low content. Unfortunately, direct spectroscopical measurement of the CBPUC electrodes themselves has proven futile, as the FTIR spectrum is oversaturated and no bands are discernible.

Instead, we have extracted the CBPUC cylinder samples with acetone, which is very volatile and miscible with DMF, as described in the experimental section. To estimate the samples’ retention of DMF, we have performed a profiled wet spinning of the same shape, but have removed samples at different intervals of time after the spinning dope coagulated in the water. Namely, immediately after coagulation, no shape could be retained, and after 30, 60, and 180 min, the spun material remained in the water undisturbed. Pure acetone and 10% DMF solution in acetone were used as controls in the FTIR measurements.

**Figure 7:**
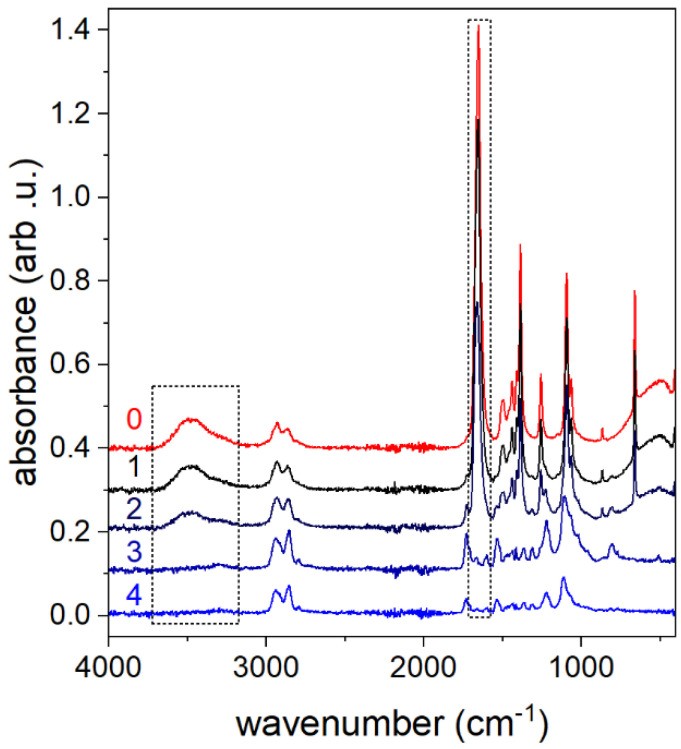
FTIR spectra of the acetone extracts of the CBPUC samples removed from coagulation bath at different times: (1) immediately after coagulation, (2) 30 min, (3) 60 min, and (4) 180 min. Curve (0) is recorded in a control experiment with 10% DMF solution in acetone. The dotted frames indicate bands particularly characteristic of the DMF.

The obtained spectra, except the acetone control, are presented in Figure 7. It can be clearly seen that the extract samples that did not have time to undergo solvent exchange, e.g., the immediately removed sample (curve 1), exhibit sharp bands characteristic of DMF (curve 0). Moreover, the FTIR data indicate that the DMF is retained in the samples that were left in water up to 30 min. After 1 h of exposure (curve 3), no DMF bands are observed. To ensure that no DMF is at all retained in the samples, 3 h of exposure to a water bath was used in the further work. These were assumed to be safe to be used in skin contact studies.

### 3.5. Performance Assessment for Transcutaneous Stimulation Use

**Figure 8:**
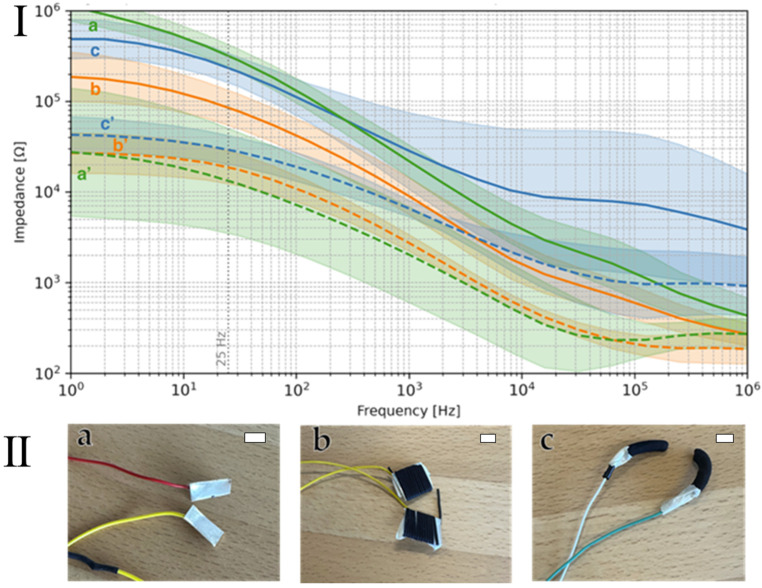
(**I**) Average impedance curves for the electrodes of various materials and shapes: (a) silver electrodes, (b) CBPUC fiber wrapped around silver, and (c) profiled elastic CBPUC electrodes. Solid curves (a–c) are impedances obtained in dry conditions; dashed curves (a’–c’) are results obtained with the application of electrolyte spray. The background shapes in light colors represent the variability of the measured impedance for respective curves. (**II**) The photographs below the graph represent the corresponding electrodes: (**a**) Ag plate, (**b**) Ag + fiber, and (**c**) profiled elastic electrodes. The scale bars are 1 cm.

Next, we evaluated CBPUC electrodes for transcutaneous stimulation (e.g., taVNS) by probing their material-to-skin impedance in the frequency domain, as well as their effective stimulation impedance in the time domain during biphasic stimulation pulses generated by a stimulator. Silver metal electrodes served as a reference standard for comparison against two different CBPUC samples produced using two different methods: single fiber wet-spinning and profiled-extrusion wet spinning. The first electrode was comprised of CBPUC fibers wrapped around a silver electrode, and the second was a hollow cylindrical CBPUC electrode (Figure 8). For brevity, we will now refer to the studied electrodes as follows. The silver plate is marked Ag plate, the CBPUC fiber wrapped around the silver plate is marked Ag + fiber, and the profiled wet spun hollow cylinder electrode is marked profiled elastic electrode.

Frequency-domain measurements revealed the expected frequency-dependent behavior of the skin–electrode interface, namely that impedance decreases with increasing frequency (e.g., as described in dynamic impedance models) (Figure 8) [38].

Under dry conditions, both CBPUC-based electrodes, Ag + fiber and profiled elastic electrode, generally exhibited lower impedance than the Ag plate, particularly at frequencies below $10^{2}$ Hz, although absolute impedances remained high (in the $10^{5}$–$10^{6}$ Ω range). Next, we applied a wetting condition (with electrolyte spray), which improved the transcutaneous impedance across all electrode types, as expected. Under wet conditions, all electrodes exhibited impedances below $10^{5}\;$Ω across the whole measured frequency range. In this regime, Ag plate and Ag + fiber showed nearly identical performance and tended to outperform the profiled elastic electrode, especially at higher frequencies above $10^{2}$ Hz.

**Figure 9:**
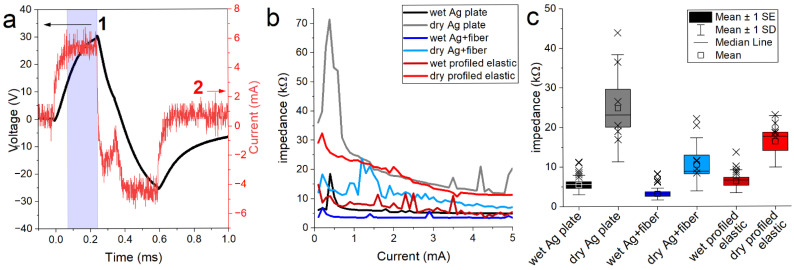
Impedance properties during biphasic current-controlled stimulation. (**a**) Example trace of applied voltage (1) and current (2) at a setting of 5 mA. The blue band denotes the area used for calculations. (**b**) Ohm’s law calculation for impedance across a range of different current settings for wet and dry electrodes. (**c**) Average impedance of different electrodes (*n* = 7). “Wet” refers to measurements performed after applying an electrolyte spray to the electrode surface, whereas “dry” refers to measurements taken without electrolyte. Wet and dry data were obtained from the same electrode types, consisting of Ag plate, Ag + fiber, and profiled elastic electrodes, as indicated in the legend (wet vs. dry shown for each material). Crosses indicate experimental data points used for construction of box plots.

Using standard taVNS parameters (biphasic current pulses, Figure 9a), we recorded voltages during the stable (second) phase of each pulse. From the relation between applied current and measured voltage, we computed electrode impedance as a function of set current (Figure 9b). As expected, higher stimulation currents yielded lower apparent impedance across all electrode types [51]. Dry measurements consistently produced higher impedance than wet; however, on average, all electrodes under wet conditions achieved impedances below ~10 kΩ. Interestingly, in dry conditions, only the Ag + fiber and profiled elastic electrodes remained consistently under the theoretical limit of ~26.67 kΩ (which permits 3 mA stimulation at 80 V, see Section 2. Materials and Methods). Figure 9c also shows the results of the impedance tests of the studied electrodes across multiple participants. The data demonstrates that the CBPUC-based electrodes show robust performance across subjects, especially compared to dry Ag plate. Moreover, the CBPUC-based electrodes demonstrated superior skin–electrode impedance characteristics compared with commercially available transcutaneous electrode materials. Marquez-Chin et al. reported average impedance values of 36.06 ± 77.70 kΩ for hydrogel electrodes, 401.19 ± 664.63 kΩ for dry polymer nanocomposite electrodes, and 970.51 ± 1933.12 kΩ for dry carbon rubber electrodes. In contrast, both dry CBPUC electrodes investigated in this study (dry Ag + fiber and dry profiled elastic) exhibited substantially lower impedances, consistently below 20 kΩ [52]. These results suggest that CBPUC-based materials may be viable for dry transcutaneous stimulation, and in many cases outperform silver, though wet (electrolyte-assisted) use remains preferable for optimal performance. It is also important to note that the arm was not intended to replicate the geometry, curvature, or tissue composition of the auricle and the results may not fully predict the performance and biocompatibility with the complex three-dimensional structure of the ear, and this remains to be further investigated.

In conclusion, the developed method seems promising for the fabrication of transcutaneous stimulation electrodes. The production technique allows for easy variability of the electrode dimensions: both length and diameter, as well as complex inner geometries. This, in its turn, opens the potential to produce several electrode shapes that could fit in many possible anatomical variations of the external ear. Ease of modification of nozzle geometries also allows for the production of customized shapes to fit well with an individual’s specific ear. Notably, the materials used in the process are cheap, conductive, and soft. Together with good electrical properties and elasticity/flexibility, the profiled electrodes become excellent candidates for future usage in taVNS in dry conditions.

## 4. Conclusions

We demonstrate a facile approach to wet spinning of conductive elastic fibers based on commercially available polyurethane and conductive filler carbon black, with the objective of producing flexible, soft electrodes for transcutaneous electrical nerve stimulation applications. These spun materials show kΩ/cm level lengthwise resistances in ~1 mm diameter fiber form, while demonstrating sub-MPa elastic moduli. We then modified this process by replacing a narrow-diameter spinneret (a blunt needle in our case) with a more complex custom-made 3D printed nozzle. A differently shaped cross-section of the nozzle allows for ‘spinning’ or ‘extrusion’ of more complex shapes, which we show with an example of a hollow cylinder. Both the conductive fiber and the hollow cylinder electrodes have shown excellent performance in conductivity under strain and compression, reaching sub-kΩ resistances under just several N of compression. The electrodes produced using this profiled wet spinning method have shown promising results when tested as materials for transcutaneous stimulation, especially in the context of dry applications where the impedance stays consistently below the theoretical limit of ~26.67 kΩ (which permits 3 mA stimulation at 80 V). This shows promise for future applications in taVNS, even if the initial tests were carried out in planar anatomical geometries. Overall, this work establishes a scalable route to fabricate soft, conductive polymer electrodes with tunable geometry and favorable electromechanical properties. The demonstrated hollow cylindrical design offers a promising platform for conformal, low-impedance interfaces in anatomically complex sites such as the external ear, supporting next-generation applications in transcutaneous neurostimulation, including taVNS.

## Figures and Tables

**Table 1:** Electrical resistances, elongation at break, and elastic moduli measured for CBPUC fibers produced by wet spinning of precursor composite with different loads of carbon black.

% *w*/*w* CB/PU Dope	Lengthwise Electrical Resistance of the Fibers, kΩ/cm	Elongation at Break, Strain %	Elastic Modulus, MPa
2	583.00 ± 93.20	289 ± 8.60	0.308
4	119.20 ± 12.67	76 ± 6.63	0.34
6	1.50 ± 0.17	55 ± 7.75	0.55
7	1.29 ± 0.06	32 ± 1.87	1.02
8	0.68 ± 0.05	11 ± 2.34	1.38
10	0.47 ± 0.05	~0	N/A

## Data Availability

The original contributions presented in this study are included in the article. Further inquiries can be directed to the corresponding authors.
